# Diverse Role of SNARE Protein Sec22 in Vesicle Trafficking, Membrane Fusion, and Autophagy

**DOI:** 10.3390/cells8040337

**Published:** 2019-04-10

**Authors:** Muhammad Adnan, Waqar Islam, Jing Zhang, Wenhui Zheng, Guo-Dong Lu

**Affiliations:** 1 State Key Laboratory of Ecological Pest Control for Fujian and Taiwan Crops, and Key Laboratory of Bio-pesticides and Chemical Biology Ministry of Education, Fujian Agriculture and Forestry University, Fuzhou 350002, China; alvi.adnan@yahoo.com (M.A.); 510772030aa@sina.com (J.Z.); 2 College of Geographical Sciences, Fujian Normal University, Fuzhou 350007, Fujian, China; ddoapsial@yahoo.com

**Keywords:** *Sec22*, ER, ERGIC, Golgi, vesicle trafficking, autophagy

## Abstract

Protein synthesis begins at free ribosomes or ribosomes attached with the endoplasmic reticulum (ER). Newly synthesized proteins are transported to the plasma membrane for secretion through conventional or unconventional pathways. In conventional protein secretion, proteins are transported from the ER lumen to Golgi lumen and through various other compartments to be secreted at the plasma membrane, while unconventional protein secretion bypasses the Golgi apparatus. Soluble *N*-ethylmaleimide-sensitive factor attachment protein receptors (SNARE) proteins are involved in cargo vesicle trafficking and membrane fusion. The ER localized vesicle associated SNARE (v-SNARE) protein Sec22 plays a major role during anterograde and retrograde transport by promoting efficient membrane fusion and assisting in the assembly of higher order complexes by homodimer formation. Sec22 is not only confined to ER–Golgi intermediate compartments (ERGIC) but also facilitates formation of contact sites between ER and plasma membranes. Sec22 mutation is responsible for the development of atherosclerosis and symptoms in the brain in Alzheimer’s disease and aging in humans. In the fruit fly *Drosophila melanogaster*, Sec22 is essential for photoreceptor morphogenesis, the wingless signaling pathway, and normal ER, Golgi, and endosome morphology. In the plant *Arabidopsis thaliana*, it is involved in development, and in the nematode *Caenorhabditis elegans*, it is in involved in the RNA interference (RNAi) pathway. In filamentous fungi, it affects cell wall integrity, growth, reproduction, pathogenicity, regulation of reactive oxygen species (ROS), expression of extracellular enzymes, and transcriptional regulation of many development related genes. This review provides a detailed account of Sec22 function, summarizes its domain structure, discusses its genetic redundancy with Ykt6, discusses what is known about its localization to discrete membranes, its contributions in conventional and unconventional autophagy, and a variety of other roles across different cellular systems ranging from higher to lower eukaryotes, and highlights some of the surprises that have originated from research on Sec22.

## 1. Introduction

**Figure 1:**
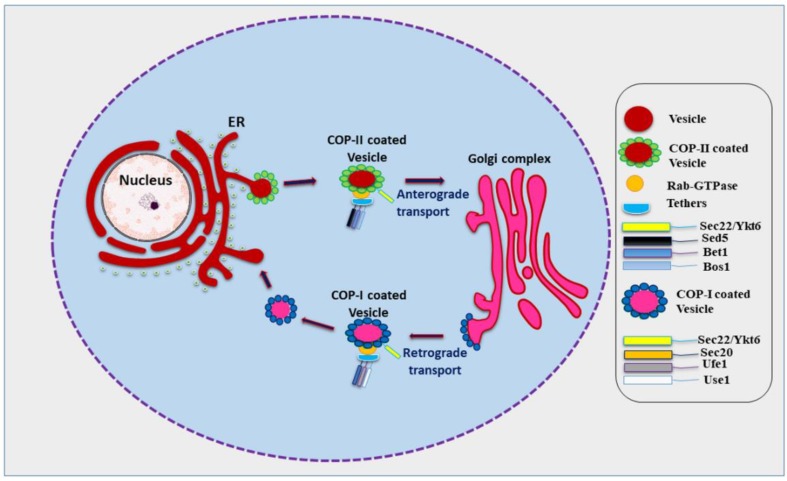
Role of SNARE proteins in anterograde and retrograde trafficking in yeast (Role of Sec22 during anterograde and retrograde trafficking along with compatible SNAREs and associated factors like COP-I (Coat Protein-I) and COP-II (Coat Protein-II), Rab-GTPase (Ras proteins related Guanosine Triphosphatase), and tethers. However, Ykt6 can replace Sec22 during retrograde trafficking from Golgi to endoplasmic reticulum (ER)).

In eukaryotes, most proteins to be secreted enter the endoplasmic reticulum (ER) lumen. Transport to the plasma membrane occurs in the form of movement of specialized containers or transport vesicles which bud from one membrane and fuse with the next membrane starting from the ER and transiting the Golgi stack en route [1]. Various kinds of vesicles thus move across the cell carrying different kinds of cargo for delivery at different membranes. This requires a biosynthetic, secretory, and endocytic protein transport system which fulfils the physiological requirements of the cell, stabilizes its internal organization, and also aides communication with the outside environment to obtain nutrients and external signals [2]. Vesicle budding and vesicle fusion are two basic processes necessary for this transport process. Budding occurs when a vesicle pinches off from a ‘donor’ membrane and fusion occurs when the membrane of the vesicle merges with the ‘acceptor’ membrane of the target [2]. Proteins destined to be secreted at the plasma membrane are transported from the ER lumen passing through the Golgi apparatus and various cellular compartments. This path is known as conventional protein secretion or the conventional secretory pathway [3]. However, recent research has found that an increasing number of leaderless proteins are secreted that bypass the ER lumen and the Golgi apparatus, revealing the existence of alternative protein secretion pathways. In addition, other unconventional routes for the secretion of soluble or transmembrane proteins and misfolding-associated proteins with initial ER localization have also been identified [4,5,6,7]. Soluble *N*-ethylmaleimide-sensitive factor attachment protein receptors (SNARE) proteins aid efficient and controllable fusion of biological membranes [8,9]. Efficient membrane bilayer fusion involves four SNARE proteins which interact via *a*-helical coil and brings the two membranes into tight proximity forcing out the water between the membranes, this trans-SNARE complex is termed a SNAREpin, while the SNAREs are referred to as v-SNAREs (bound to vesicle membrane) and t-SNAREs (bound to target membrane) [8,10]. Within the coiled coil of the SNAREpin, surface contact between the helices mainly depends on hydrophobic interactions, with the exception of an ionic layer in the middle of the coiled bundle [11,12]. Interestingly, one R-SNARE interacts with three Q-SNAREs, named after their interacting residues arginine (R) or glutamine (Q) [13,14]. SNAREs are located on plasma membrane, lysosomes/vacuoles, Golgi membranes, endoplasmic reticulum, and the vesicles derived from each of these membranes [15,16]. Sec22 is an important R-SNARE involved in membrane fusion in Eukaryotes. Sec22 localizes to ER and Golgi and helps in anterograde and retrograde transport of vesicles [17,18,19]. In anterograde transport, cargo vesicles bud off from the ER; these vesicles are coated with coat protein–II (COP-II) and involve various other factors including Rab-GTPase, tethers, a set of Q-SNAREs, and the R-SNARE Sec22 (Figure 1) [20,21,22]. The SNAREs of COP-II coated vesicles will interact with the SNAREs of cis Golgi resulting in membrane fusion and exchanges of vesicular components [23]. Here, Sec22 acts as a vesicle SNARE (v-SNARE) and combines with its respective target SNARE (t-SNAREs) in the cis Golgi. In retrograde transport, cargo vesicles move in the opposite direction from the Golgi towards the ER in a similar fashion as in anterograde transport (Figure 1) [24,25,26]. During retrograde transport, COP-I, upon activation of Arf1 (ADP-ribosylation factor 1) GTPase, will be recruited to the Golgi membrane which leads to sorting and assembly of cargo proteins into a cage like structure followed by budding off of the vesicles from the Golgi membranes or ER–Golgi intermediate compartment (ERGIC) [24,27]. Sec22 homodimers promote assembly of higher order SNARE-complexes to catalyze this membrane fusion [9,19]. The role of Sec22 is not only confined to ERGIC but it is also needed in the formation of contact sites between the ER and the plasma membrane [19,28,29,30].

## 2. Protein Structure of Sec22

**Figure 2:**
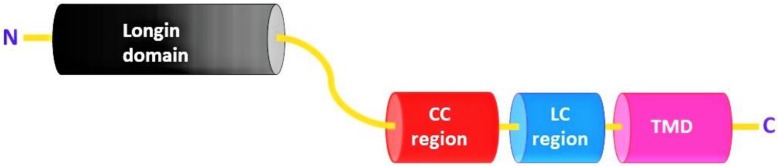
Schematic representation of Sec22 with N-terminal Longin domain, coiled-coil region (CC), low-complexity region (LC), and its C-terminal transmembrane domain (TMD).

**Figure 3:**
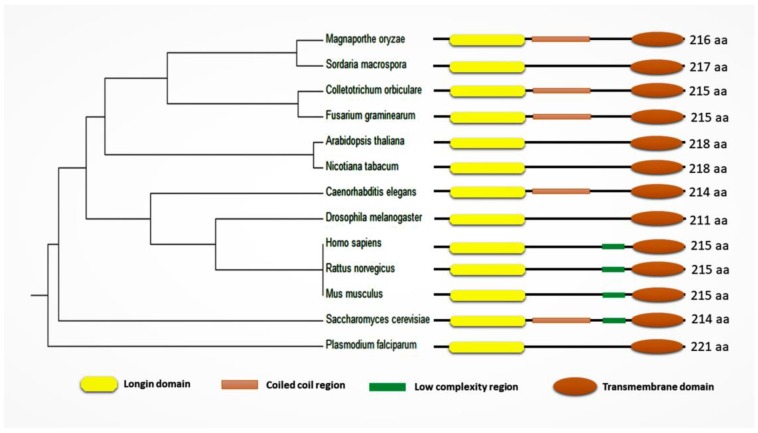
Phylogenetic analysis and domain characterization of Sec22 and its homologues (Sec22 of *Saccharomyces cerevisiae* and its homologues among different organisms including *Homo sapiens*, *Mus musculus*, *Rattus norvegicus, Drosophila melanogaster*, *Caenorebditis elegans*, *Plasmodium falciparum*, *Arabidopsis thaliana*, *Nicotiana tabacum*, *Magnaporthe oryzae*, *Colletotrichum orbiculare*, *Sordaria macrospora*, and *Fusarium graminearum*). Longin domain is a conserved N-terminal domain with a profilin-like fold which is considered an essential regulator. The coiled-coil region is important in soluble *N*-ethylmaleimide-sensitive factor attachment protein receptor (SNARE) zippering. The low-complexity region may be involved in flexible binding associated with specific functions. The transmembrane domain is a typical stretch of hydrophobic residues located at the C-terminus, involved in anchoring the protein to the membrane and participating in other aspects of the functions of these proteins.

Sec22 protein consists of a Longin domain, a coiled-coil region, a low-complexity domain, and a transmembrane domain. Yeast (*Saccharomyces cerevisiae*) Sec22 is a 214 amino acid (aa) long protein structure and has all four domains (longin domain (33–116 aa); coiled-coil region (127–150 aa); low-complexity region (159–172 aa); and a transmembrane domain (189–211 aa)). Most of these conserved domains are involved in protein function and localization (Figure 2) [19]. Similarly, *Sec22* homologues among different organisms all contain a longin domain along with the transmembrane domain. However, the low-complexity region and the coiled-coil region appear not to be conserved in some of the orthologs (Figure 3) (Table S1).

## 3. Role of *Sec22* in Yeast

Yeast (*S. cerevisiae*) is considered a highly useful model for illustrating the function of the components involved in membrane fusion. These components have been identified through bioinformatics, biochemistry, and genetics, and all of the described SNAREs are present [31]. Physical interaction of Sec22 with ER to Golgi SNARE proteins illustrates its role in anterograde trafficking; however, it also participates with Sec20 and Ufe1 proteins for retrograde trafficking [29,32,33,34,35,36]. How the functional domains of Sec22 direct this protein from the ER to Golgi membranes and form the SNARE complex with Sed5, Bos1, and Bet1 was investigated by Liu and colleagues [37]. They characterized the behavior of an array of Sec22 deletion mutants in COP-II budding assays, SNARE complex immunoprecipitations and subcellular fractionation gradients. It was shown that the Sec22 N-terminal profilin-like domain was important but not sufficient for COP-II dependent export of Sec22 from the ER. Sec22 lacking the N-terminal domain was assembled in the ER/Golgi SNARE complex but could not export from the ER. However, some of Sec22 mutants were properly packaged into COP-II vesicles but failed to assemble in the SNARE complex [37]. Thus, we can assume that Sec22 exports from the ER, packaging into COP-II transport vesicles and subsequent targeting to the Golgi complex may be independent of SNARE pairing. Another study shows that Sec22 binds to its respective SNARE partners, since Sec22 and NYV1 can replace Snc1 and Snc2 in vitro but cannot replace them in vivo as they are confined to their respective compartments. This suggests strict requirements of SNAREs targeting particular compartments enhancing specificity of intracellular membrane fusion/attachment events [38,39].

Mutation of Sec22 along with Tip20 appears to be lethal for *S. cerevisiae* because both of these proteins are required for vesicular transport between the ER and Golgi complex [40]. Tip20 is a cytoplasmic protein bound to the surface of the ER and is responsible for binding or un-coating of COP-I coated vesicles during retrograde transport. COP-I coated vesicles are involved in quick recycling of Sec22 from Golgi to the ER, since Sec22 behaves like an ER resident protein [41,42]. The SNARE protein Sec22 contains a motif that binds with the COP-II sub-complex (Sec23/24) and specifies its ER exit as an unassembled SNARE. The crystal structure of Sec22 bound to Sec23/24 reveals that the transport signal is a folded epitope rather than a conventional short peptide signal sequence. During this process, the NIE segment (refers to conserved N-I-E sequence) of the SNARE motif folds against the N-terminal longin domain. This closed form of Sec22 binds at the Sec23/24 interface. Thus, COP-II recognizes unassembled Sec22 via a folded epitope, whereas Sec22 assembly into SNARE complexes would mask the NIE segment. The concept of a conformational exposed epitope as a transport signal suggests a packaging mechanisms in which a coat is sensitive to the folded state of a cargo protein or the assembled state of a multiprotein complex [43].

It was generally believed that *Sec22* mutations cause defects both in anterograde transport and retrograde transport [44,45]. However, later studies proved that *Sec22* mutation could not affect anterograde transport and only retarded retrograde transport. Thus, it would be interesting to know how the early secretory pathway is sustained in the absence of Sec22. Fusion of ER derived vesicles and Golgi acceptor membranes require Sec22 involvement; however, Ykt6, a related R-SNARE, which comes into play during later stages of the secretory pathway is not only upregulated but functionally compensates for Sec22 by forming a SNARE complex with Sed5 and Bos1 [13,33,46]. Under such circumstances, Ykt6 not only packages properly onto the ER export vesicles but is also considered essential for vesicle fusion with the Golgi. Ykt6 overexpression could rescue Sec22 deficiency in yeast but these proteins do not interact in vivo as Sec22 and Ykt6 belong to the same R-SNARE class; therefore, a functional participation of both these SNAREs during membrane fusion within the same SNAREpin is considered unlikely [13,46,47,48] as they belong to two separate SNAREpins. Ykt6 is generally associated with vacuolar SNAREs where it competes with Nyv1 (a vacuolar v-SNARE) for binding with vacuolar t-SNAREs [13]. This indicates the presence of two separate SNARE complexes. However, we can assume that the requirements of specific SNARE proteins in intracellular membrane fusion are not as strict as expected and this suggests collaborative mechanisms using both SNARE proteins and upstream elements to maintain a significant level of compartmental organization.

Here, an interesting question arises! If Sec22 is required for retrograde transport from Golgi to ER, could Ykt6 possibly also substitute for Sec22? Could it thus yield a SNARE complex for retrograde transport and fusion? We can speculate that Ykt6 fulfills this requirement but then other characterized SNARE proteins of the retrograde pathway such as Bos1, Ufe1, and Bet1 would also be crucial. Similarly, mammalian homologs of Bet1 (rbet), Sed5 (syntaxin 5), Bos1 (membrin), Ykt6 (Ykt6), and Sec22 (Sec22b) have been found to be functionally associated with retrograde transport in mammals [49]. Another glaring difference between Sec22 and Ykt6 is that Sec22 is tail anchored via transmembrane domain (TMD) and plays a role during retrograde and anterograde trafficking while Ykt6 is lipid anchored and plays roles in trafficking at vacuoles, endosomes, and Golgi. Although Sec22 and Ykt6 have similar longin domains, Sec22 has an open conformation compared to Ykt6, which can also adopt a closed conformation [50]. It can be difficult to compare mammals and yeast as there are various isoforms of Sec22 in mammals that localize to different compartments, and the organization of the early secretory pathway seems to vary between species. As Ykt6 can compensate for Sec22 and yield a SNARE-complex for the retrograde fusion, we can conclude that the presence of a parallel transport pathway in the absence of Sec22 is unlikely as this results in growth-rate inhibition and reduction in transport activity [46,51]. Additionally, double mutation of both of *Sec22* and *Ykt6* is lethal indicating that the presence of Ykt6 in the absence of *Sec22* is absolutely necessary.

Sec22, along with Sey1 (dynamin-like GTPase of atlastin family), is required for homotypic ER membrane fusion, illustrating a role for Sec22 in membrane fusion of higher order complexes [52,53,54]. Sec22, Sey1, and Rtn1 are considered essential for normal ER morphology. Single gene deletions of these proteins do not produce considerable ER morphological changes but double gene deletion of *Sec22* and *Sey1* or *Sec22* and *Rtn1* result in abnormal ER morphology. Irregular ER morphology as a consequence of these gene deletions is marked by the absence of tubular networks and the presence of expanded ER sheets [52,53,54]. *Sec22* mutation causes growth defects in yeast, and these defects can be suppressed with the overexpression of Bos1/Bet1 proteins (anterograde transport SNARE proteins). *Sec22* and *Bet1* double mutation is as lethal as *Sec22* and *Ykt6* double mutation, which result in considerable growth reduction [55,56].

## 4. Filamentous Fungi

SNARE protein functions in secretory and other processes have been described for filamentous fungi. The role of *Sec22* in *M. oryzae, C. orbiculare, S. macrospora, Verticillium dahliae*, and *F. graminearum* have been demonstrated. *M. oryzae* require appropriate developmental conditions for conidiogenesis and appressorium formation during rice blast disease establishment (Table 1) [57]. Song and colleagues found that deletion of *MoSec22* resulted in reduced conidiation and appressorium formation as both these processes are regulated by membrane trafficking [58]. Loss of *Mosec22* causes the inability to produce conidiophores due to autolysis of aerial hyphae that support conidiophore differentiation and asexual spore production [58,59,60]. Moreover, deletion of *Mosec22* increases cell wall sensitivity to cell wall stressors and results in abnormal accumulation of chitin [58,61]. Fungal polarized growth requires endocytic uptake and recycling of cell wall proteins such as chitin synthases. Deletion of *MoSec22* blocks endocytosis and reduces re-uptake of chitin synthases which impairs polarized growth. *MoSec22* is also important for regulating reactive oxygen species (ROS) availability, which is considered essential for *M. oryzae* during rice blast disease establishment [62].

We have encountered quite similar outcomes in *F. graminearum* (pathogen causing fusarium head blight (FHB) of wheat and barley), as deletion of *FgSec22* caused a plethora of problems, from vegetative growth to sexual reproduction. Interestingly, loss of *FgSec22* reduced both sexual and asexual spore production and greatly decreased the amount of pathogenicity related deoxynivalenol (DON) toxin production (unpublished data). Boenisch and colleagues have confirmed that DON enhances FHB disease severity and the majority of DON producing proteins are ER localized [63]; we therefore propose that FgSec22 could be involved in DON-related protein trafficking in *F. graminearum*. Similarly, *C. orbiculare* (hemibiotrophic pathogen of cucumber) expresses different virulence related effectors at biotrophic and necrotrophic stages. The secretion of these effectors via the ER/Golgi route and subsequent exocytosis towards the host–pathogen interface involves Sec22 [64]. In *S. macrospora*, the *Sec22* mutant exhibited reduced numbers of ascospores, as well as defects in their pigmentation, maturation, and germination. The *S. macrospora* mutation of *Sec22* also leads to transcriptional deregulation of many development related genes and the majority of these genes encode proteins which are associated with the ER or vesicle dependent transport [65]. Recently, deletion of a *Sec22* homologue in *V. dahliae*, *VdSec22*, was shown to result in reduced virulence [66]. *VdSec22* regulates secretion of many enzymes, such as cellulases, xylanases, and pectinases, involved in carbohydrate hydrolysis and cell wall degradation of the plant host [67]. All of these studies suggest the significant role of Sec22 in the trafficking of virulence related proteins from the ER.

## 5. Plants

In *A. thaliana*, loss of *Sec22* results in impaired gametophyte development as pollens become abnormal during the bi-cellular stage [68]. In addition, the majority of embryo sacs could not support embryogenesis and their central cell displayed unfused polar nuclei, as well as Golgi consumption and fragmentation due to loss of *Sec22* [68]. Similarly, overexpression of *Sec22* and *Memb11* induced collapse of Golgi membrane proteins into the ER in *N. tabacum* [69]. In addition, Sec22 along with other factors helps in the ER export of Phyl1.1 (Phytolongin) which plays important roles during vesicle formation and vesicle fusion in plants [70,71].

## 6. Mammals

The SNARE complex involved in the fusion of ER derived COP-II vesicles and Golgi membrane include v-SNARE Sec22 and t-SNAREs Syntaxin-5, Bet1 and membrin; while the SNARE complex mediating fusion of Golgi-derived COP-I vesicles and the ER involve SNARE proteins Sec22, Use1, Sec20, and Syntaxin-18 [72,73,74,75]. Mammalian cells have *Sec22a* and *Sec22c* homologues in addition to *Sec22b* [76,77]. Of the three mammalian Sec22s, msec22b (*Sec22* homologue from mouse), most closely resembles yeast Sec22 with respect to length and hydrophobicity profile; however hsec22c (human) seems more similar to rsec22a (rat) in these features. Although, hsec22c is more ubiquitously expressed compared rsec22a in terms of tissue expression pattern. Its localization pattern is generally similar to msec22b and rsec22a, which are present at the ER/Golgi boundary [78,79,80,81]. Overexpression of both hsec22c and msec22b perturb the localization of ER/Golgi SNARE molecules by interacting with the components of the transport machinery of the early secretory pathway [79]. This suggests that both of them function in the same region. But why do mammalian cells require three Sec22 proteins compared to only one in yeast? We know that yeast Sec22 participates both in anterograde and retrograde transport and forms SNARE complexes at both the ER and Golgi ends. The function of each of the mammalian Sec22s may be much more specific by participating either during anterograde or retrograde transport by establishing Sec22 variety specific SNARE complexes at each end.

Generally, Sec22 SNARE proteins are tail-anchored proteins with single TMD at the C-terminus whereas mammalian Sec22 proteins may have multiple TMDs [28,77]. The TMD of Sec22 is required for efficient homodimer formation which promotes assembly of higher order SNARE complexes by catalyzing membrane fusion [19]. Sec22 engages in efficient homodimer formation with cellular membranes when cysteine residues are positioned at the C terminus or SNARE motif of TMD. The presence of specific Sec22 cysteine derivatives, both on donor COP-II vesicles and acceptor Golgi membranes, and the formation of disulfide crosslinks provide clear readouts on *cis* and *trans* SNARE arrangements during this fusion event. Yamamoto and colleagues generated several Sec22 isoforms by alternate splicing and changed the number of TMDs at the C-terminus in order to determine their physiological significance [76]. Splicing isoforms with four TMDs were unexpectedly localized at the cis Golgi while isoforms having fewer than four TMDs were ER localized. Therefore, sub-cellular localization of Sec22 can be altered as a result of changes in the number of TMDs, which ultimately affects the function of Sec22.

Sec22 is important for proper protein secretion from the ER as it forms a complex with the ER resident protein Slt1; however, downregulation of Slt1 results in improper secretion of proteins [48,82]. Sec22b plays a role as a v-SNARE in VLDL (very-low-density lipoprotein)-transport vesicle (VTV) fusion with the Golgi. VLDLs play a critical role in atherosclerosis and VLDLs are prepared in the hepatic ER and transported to Golgi by VTV [83]. Zhao and colleagues found that Sec22b takes part in protein transport and regulates cell motion [84]. Similarly, Sec22b has been associated with protein trafficking and translocation while its downregulation in the hippocampus as seen in aging and the brains of Alzheimer’s patients (Table 1) [85,86]. Sec22b has a physiological role during neuronal development and brings plasma membrane in close proximity to ER by interacting with the t-SNARE syntaxin 1A [87]. Overexpression of the longin domain of Sec22b in developing neurons reduces neurite outgrowth [87]. However, the exact role of the longin domain of Sec22b in this process remains to be determined.

## 7. Other Organisms

### 7.1. Drosophila Melanogaster

The absence of *Sec22* causes ER proliferation, an expanded ER lumen, abnormal Golgi morphology, and enlargement of late endosomes in *D. melanogaster* (Table 1) [88,89]. Mosaic analysis of the *Drosophila* eye illustrated that *Sec22* mutants have small rhabdomeres which are sometimes fused with each other. Photoreceptor cells alos have a highly expanded ER morphology which is gradually lost with aging [17]. Therefore, Sec22 is not only considered important for vesicular trafficking but also for normal ER morphology and eye morphogenesis in *D. melanogaster*. Sec22 is crucial for the wingless (Wg)/Wnt signaling pathway in *D. melanogaster* (Table 1). Wnt signaling plays a significant role in development, tissue homeostasis, and during disease. Proper regulation of Wnt secretion is therefore crucial in wingless signaling in which transport cargoes are prepared by assembly and packaging of multiple molecular machines. In this pathway, protein p24 interacts with Wg which acts as cargo selector and packages Sec22 with Wg for anterograde transport [18].

### 7.2. Caenorhabditis Elegans

In *C. elegans*, Sec22 is involved in importing of RNAi silencing signals or cell autonomous RNA interference (RNAi) and interacts with RNA transport protein Sid5 associated with late endosomes (Table 1) [90]. Small RNA pathways, including RNAi, regulate gene expression, and although RNAi is primarily considered to be cytoplasmic, the processes are also related to membranes. Effective RNAi requires maturation of late endosomes/multivesicularbodies (MVBs); however, fusion of MVBs to lysosomes decreases silencing efficiency as Sec22 localizes to MVBs/late endosomes. Mutation of *Sec22* causes an increase in RNAi efficiency upon double-stranded RNA ingestion while the levels of RNAi activity decline with overexpression of *Sec22* [90].

### 7.3. Plasmodium Falciparum

The distinct mechanism of Sec22 trafficking in the malarial parasite, *P. falciparum*, was studied by Ayong and colleagues [91]. In order to understand the machineries and signals during SNARE protein targeting of respective intracellular locations, the *Sec22* ortholog in *P. falciparum* (*PfSec22*), with an atypical insertion of a *Plasmodium* export element in the N-terminal domain, was studied. Sec22 protein was associated partially with membrane structures of parasitized erythrocytes. They found that an atypical longin domain encompasses signals for ER/Golgi recycling of PfSec22 and fractional export beyond the ER/Golgi interface. They suggested that the PfSec22 longin domain displays clear differences from yeast and mammalian orthologs and shows discrete Sec22 trafficking in malarial parasites [91].

The above findings from yeast, filamentous fungi, mammals, plants, *Drosophila*, *C. elegans*, and *P. falciparum* indicate that the molecular function of *Sec22* can be conserved, but with species-specific roles in various organismic and cellular processes among eukaryotes.

## 8. Role of *Sec22* in Autophagy

Autophagy, is a catabolic process involved in cellular homeostasis by degrading cytoplasmic proteins and organelles. Dysregulation or failure of autophagy can result in various problems such as inflammation, cancer, and neurodegeneration [94,95,96]. Autophagy initiates with the formation of small cup-shaped membraned structures called phagophores; their edges elongate and engulf proteins and/or organelles and form double-membraned autophagosomes after fusion. Thus, autophagosome formation causes the sequestration of cytoplasmic portions and transports these to lysosomes containing hydrolases for degradation of the autophagic cargoes [95,97,98,99]. The membrane source for autophagosome genesis has been intensely debated over the last four decades and it is generally considered that autophagosomes derive their membrane source from the plasma membrane, Golgi, ER, and mitochondria [100,101,102,103,104,105]. During this time, it was also believed that SNAREs have no significance during autophagosome synthesis and that the elongation of the phagophore depends on de novo lipid addition. Nevertheless, new findings suggest several roles for SNAREs in autophagy both in the genesis of autophagosomes and autophagosome–lysosome fusions [106,107].

SNAREs regulate autophagosome formation, and homotypic fusion of phagophore precursors is regulated by the SNAREs VTI1B, Syntaxin-17, Syntaxin-18, and VAMP-7 in mammals [108,109,110,111]. Owing to such fusion events, these structures develop into a tubular network leading to the formation of phagophores and autophagosomes [108]. SNAREs which are involved in exocytosis are very important for autophagosome formation in yeast. These SNAREs regulate the synthesis of tubulovesicular structures, which are positive for Atg9 (transmembrane protein necessary for autophagy) [109]. Lack of these SNAREs obliterates tubular network formation and results in small Atg9 containing vesicles, which stops homotypic fusion events [109,112]. Although Sec22 is not directly involved in exocytosis, it participates in autophagosome–vacuole fusion and regulation of tubular network formation of Atg9 in that Sec22 enables Atg9 recruitment to the phagophore assembly site [29,109].

Sec22 also plays a significant role in the unconventional secretion of leaderless cytosolic proteins by secretory autophagy. Cytosolic proteins which lack leader peptides cannot enter the ER lumen but can be secreted unconventionally by exocytosis of post Golgi vesicles [7]. Kimura et al. [30] described that the prototypical secretory autophagy cargo 1L1B/1L-1 (proinflammatory cytokine or interleukin 1 beta) is initially recognized by TRIM16 which later interacts with Sec22b. TRIM16 and Sec22b jointly deliver 1L1B cargo to MAP1LC3B-II positive sequestration membranes. Sec22b together with syntaxins at the plasma membrane finally concludes the cargo secretion. However, secretory autophagy differs from normal autophagy in ways that are not fully understood, but employs particular receptors as well as dedicated SNARE machinery to circumvent lysosomal fusion.

## 9. Future Prospects

Sec22 plays a major role in vesicle trafficking and membrane fusion, which are essential processes for normal cellular functions and homeostasis. The majority of protein synthesis starts at the ER and requires highly dynamic ER orientation and tightly regulated ER morphology. This in turn requires Sec22 for the normal structure and efficient working of the ER and related organelles. Sec22 is involved in numerous pathways in filamentous fungi important for cell wall integrity, reproduction, pathogenicity, and expression of extracellular enzymes. How deletion of *Sec22* deregulates various development related genes requires further investigation. We have observed that deletion of *FgSec22* results in a 90% reduction in the production of the trichothecene mycotoxin DON encoded by TRI genes in *F. graminearum*. FgSec22 subcellular localization also coincides with the localization of TRI proteins (unpublished data); but its role in transport of DON containing ER derived “toxisomes” has yet to be explored. Overexpression of native *S. cerevisiae* ER-to-Golgi SNARE genes including *Sec22* increase heterologous cellulase secretion, therefore we assume that overexpression of *Sec22* could also result in enhanced growth, reproduction, and enzyme production in yeast and filamentous fungi. Enhanced enzyme production conceivably could increase pathogenicity of filamentous fungi. In any case, the overproduction of enzymes would be of great interest for the industrial production of enzymes, plant pathology, and the paper and pulp industry.

In *Arabidopsis*, deletion of *Sec22* results in unfused polar nuclei which leads to degenerative pollens and renders its life cycle incomplete; however, to characterize how Sec22 helps nuclear fusion during gametophyte development requires further work. Because a loss mutation of *Sec22* increases RNAi, we propose that its presence decreases RNAi as a result of its effect on MVBs or endosomal fusion with lysosomes, which degrades their cargo by lysosomal hydrolases. Loss of *Sec22* could be sustained somehow but combined loss of *Sec22* with other genes, such as *Ykt6*, *Tip20*, *Bet1*, *Sey1*, and *Rtn1*, proves fatal; this suggests that *Sec22* carries out different processes in collaboration with other genes. The membrane source for autophagosome formation during conventional autophagy is derived from the plasma membrane, Golgi, and ER; all of these have some sort of interaction with Sec22; however, it is not clear whether the formation and elongation of small cup-shaped phagophores either involves Sec22 or depends on de novo lipid addition. The potential role of Sec22 beyond ER/Golgi trafficking during exocytosis of leaderless proteins raises many questions and sparks a new debate on its function in both conventional and unconventional protein secretion.

## Figures and Tables

**Table 1:** Role of *Sec22* in different organisms.

Organism	Functions of Sec22 (Other than Anterograde and Retrograde Trafficking)	References
*Saccharomyces cerevisiae*	Uptake of caesium ions, cellulase secretion, maintenance of ER morphology and autophagy	[54,92,93]
*Sordaria macrospora*	Effects sexual, asexual reproduction, regulation of ER associated proteins, melanin biosynthesis, and development related genes	[65]
*Colletotrichum orbiculare*	Transport of virulence related effectors	[64]
*Fusarium graminearum*	Effects sexual, asexual reproduction and pathogenicity	(unpublished data)
*Magnaporthe oryzae*	Effects cell wall integrity, growth, reproduction, pathogenicity, chitin deposition, regulation of reactive oxygen species (ROS) level, endocytosis, expression of extracellular enzymes	[58]
*Verticilium dahliae*	Regulates enzymatic cell wall degradation and pathogenicity	[66]
*Arabidopsis thaliana*	Gametophyte development and uptake of caesium	[92]
*Nicotiana tabacum*	Overexpression causes collapse of Golgi membrane proteins into ER	[71]
*Drosophila melanogaster*	Eye morphogenesis, wingless signaling pathway, *Sec22* mutation causes abnormal ER, and Golgi morphology	[17,18]
Mammals (*Homo sapiens, Rattusnorvegicus, Mus musculus*)	Autophagy, regulate cell motion, protein trafficking, translocation, and downregulation in the hippocampus of aging and Alzheimer’s disease brains	[72,84,86]
*Caenorebditis elegans*	Regulates RNAi	[90]
*Pasmodium falciparum*	Encompasses signals for ER/Golgi recycling and fractional export beyond the ER/Golgi interface	[91]

## Supplementary Materials

The following are available online at https://www.mdpi.com/2073-4409/8/4/337/s1.
