# Supplementary material for: Diverse Role of SNARE Protein Sec22 in Vesicle Trafficking, Membrane Fusion, and Autophagy
Source: Cells. 2019 Apr 10;8(4):337. doi: 10.3390/cells8040337 (PMC6523435; doi:10.3390/cells8040337)
Supplement: Supplementary file 1 [file cells-08-00337-s001.pdf]

Table S1: Sec22 SNARE homologues used during this study

Organism	**E-Value**	**NCBI reference number**	**Longin domain (aa)**	**Coil coiled region (aa)**	**Low complexity region (aa)**	**TMD length (aa)**
Saccharomyces cerevisiae	8e-161	NP_013370.1	33-116	127-150	159-172	189-211
Sordaria macrospora	9e-163	XP_003346158.1	34-119			197-216
Colletotrichum orbiculare	4e-149	BAO27797.1	34-118	129-152		191-213
Fusarium graminearum	7e-163	XP_011323737.1	34-118	129-151		191-213
Magnaporthe oryzae	2e-161	XP_003719791.1	34-119	130-153		192-214
Arabidopsis thaliana	1e-165	NP_172653.1	35-119			193-212
Nicotiana tabacum	7e-165	XP_016484743.1	35-119			193-215
Drosophila melanogaster	5e-159	NP_569863.1	36-118			189-210
Homo sapiens	3e-162	NP_004883.3	36-118		162-179	191-213
Rattus norvegicus	1e-162	NP_001020857.1	36-118		162-179	191-213
Mus musculus	2e-162	NP_035472.1	36-118		162-179	191-213
Caenorebditis elegans	7e-161	NP_508198.1	37-118		160-186	191-312
Pasmodium falciparum	7e-162	XP_001351271.1	31-123			194-216

*Blue color represents absence of coil coiled region or low complexity region

Organism	**Functions of Sec22**	Sec22 interacting proteins	Function of Sec22 interacting proteins	References
Saccharomyces cerevisiae	Anterograde and retrograde trafficking, uptake of caesium ions, cellulase secretion, maintenance of ER morphology, autophagy	Bet1, Bos1, Sed5, Sec20, Ufe1, Slt1, Snc1/2, Sey1, Rtn1 Tip20, atg9,	Bet1, Bos1, Sed5 collaborate with sec22 during anterograde transport. Bos1 or Bet1 overproduction suppresses the secretory and growth defects of Sec22 mutant. Sec20, Ufe1, Slt1, Snc1/2 the ER proteins cooperate with Sec22 in the retrograde Golgi to ER trafficking.<br>Sey1, and Sec22 are involved in the homotypic ER membrane fusion.<br>Sey1, Rtn1 and Sec22 are involved in the normal ER morphology. Tip20 could be responsible for binding or uncoating of COPI coated retrograde transport vesicles. A subset of *tip20* mutants was found to be lethal in combination with Sec22.<br>Sec22 enables atg9 recruitment to the phagophore assembly site and helps in autophagosome	(Newman et al., 1990; Lewis and Pelham, 1996, Lewis et al., 1997, Cosson et al., 1997; Frigerio, G., 1998; Tsui et al., 2001; Liu and Barlowe, 2002; Liu et al., 2004; Hu et al., 2009; Nair et al., 2011; Anwar et al., 2012; Dräxl et al., 2013; Lee, et al., 2015; Van Zyl et al., 2016)

			formation during autophagy.	
Sordaria macrospora	Effects vegetative growth, ascospore production, maturation, pigmentation, and germination. melanin biosynthesis, ER associated proteins and development related genes regulation	Melanin biosynthesis genes, development related genes		(Traeger and Nowrousian, 2015).
Colletotrichum orbiculare	Transport of virulence related effectors.			(Irieda et al., 2014).
Fusarium graminearum	cell wall integrity, mycelial growth, conidial production, infection of the host plant, endocytosis (unpublished data)			(Boenisch et al., 2017)
Magnaporthe oryzae	cell wall integrity, mycelial growth, conidial production, infection of the host plant, normal chitin deposition, regulation of ROS level, endocytosis, efficient expression of the extracellular enzymes peroxidases and laccases	Need to study song et al paper		(Song et al., 2010).
Arabidopsis thaliana	Gametophyte development. Regulation of uptake of non-			(El- Kasmi et al., 2011; Dräxl et al., 2013).

	essential cation caesium (Cs þ)			
Nicotiana tabacum	Overexpression causes collapse of Golgi membrane proteins into ER	Memb11, Phytolongins (Phyl)	Phyl1.1 export from the ER involves Sec22 along with other factors.	(Chatre et al., 2005; de Marcos Lousa et al., 2016)
Drosophila melanogaster	Its mutation causes ER proliferation, expanded ER lumens, abnormal Golgi morphology, enlargement of late endosomes. Essential for eye morphogenesis and Wingless (Wg)/Wnt signaling pathway	Wingless/Wnt secretion	Wingless (Wg)/Wnt signaling plays significant role in development, tissue homeostasis, and disease.	(Zhao et al., 2015; Li et al., 2015).
Mammals (*Homo sapiens*, *Rattus norvegicus*, *Mus musculus*)	Membrane fusion, autophagy, regulate cell motion. Protein trafficking, translocation, and downregulation in the hippocampus of aging and Alzheimer's disease brains.	Slt1 Use1, Sec20, Syntaxin-18	Slt1 plays role in efficient protein secretion ER. Use1, Sec20, Syntaxin-18 play role in retrograde transport	(Burri et al., 2003; Dilcher et al., 2003; Chagoyen et al., 2006; Malsam and S ӧllner, 2011; Berchtold et al., 2013; Zhao et al. 2016).
Caenorebditis elegans	Plays crucial role during import of RNAi silencing signals or cell autonomous RNAi	Sid5	Sec22 interacts with RNA transport protein Sid5 associated with late endosome	(Zhao et al., 2016).
Pasmodium falciparum	Encompasses signals for ER/Golgi recycling and fractional export beyond the ER/Golgi interface			(Ayong et al., 2009).

Organism	Functions of Sec22	Reference
Saccharomyces cerevisiae	Anterograde and retrograde trafficking, uptake of caesium ions, cellulase secretion, maintenance of ER morphology, autophagy	
Sordaria macrospora	Effects vegetative growth, ascospore production, maturation, pigmentation, and germination. melanin biosynthesis, ER associated proteins and development related genes regulation	
Colletotrichum orbiculare	Transport of virulence related effectors.	
Fusarium graminearum	cell wall integrity, mycelial growth, conidial production, infection of the host plant, endocytosis (unpublished data)	
Magnaporthe oryzae	cell wall integrity, mycelial growth, conidial production, infection of the host plant, normal chitin deposition, regulation of ROS level, endocytosis, efficient expression of the extracellular enzymes peroxidases and laccases	
Arabidopsis thaliana	Gametophyte development. Regulation of uptake of non-essential cation caesium (Cs þ)	
Nicotiana tabacum	Overexpression causes collapse of Golgi membrane proteins into ER	
Drosophila melanogaster	Its mutation causes ER proliferation, expanded ER lumens, abnormal Golgi morphology, enlargement of late endosomes. Essential for eye morphogenesis and Wingless (Wg)/Wnt signaling pathway	
**Mammals (*Homo sapiens, Rattus norvegicus, Mus musculus*)**	Membrane fusion, autophagy, regulate cell motion. Protein trafficking, translocation, and downregulation in the hippocampus of aging and Alzheimer’s disease brains.	
Caenorebditis elegans	Plays crucial role during import of RNAi silencing signals or cell autonomous RNAi	

Pasmodium falciparum	Encompasses signals for ER/Golgi recycling and fractional export beyond the ER/Golgi interface	

**Sec22 interacting proteins**	**Function**	**References**
Bet1	Bet1, Bos1, Sed5 collaborate with sec22 during anterograde transport. Bos1 or Bet1 overproduction suppresses the secretory and growth defects of Sec22 mutant.	
Bos1	Bet1, Bos1, Sed5 collaborate with sec22 during anterograde transport. Bos1 or Bet1 overproduction suppresses the secretory and growth defects of Sec22 mutant.	
Sed5		
Sec20	Sec20, Ufe1, Slt1, Snc1/2 the ER proteins cooperate with Sec22 in the retrograde Golgi to ER trafficking	
Ufe1		
Slt1		
Snc1/2		
Sey1	Sey1, and Sec22 are involved in the homotypic ER membrane fusion. Sey1, Rtn1 and Sec22 are involved in the normal ER morphology.	
Rtn1		
Tip20	Tip20 could be responsible for binding or uncoating of COPI coated retrograde transport vesicles. A subset of *tip20* mutants was found to be lethal in combination with Sec22. Sec22 enables atg9 recruitment to the phagophore assembly site and helps in autophagosome formation during autophagy.	
atg9		
Melanin biosynthesis genes, development related genes		

Memb11,		
Wingless/Wnt secretion		
Syntaxin-17		
Syntaxin-18		
Sid5		
Phytolongins (Phyl)		

Table 1. The complete set of yeast SNAREs. Each of the yeast SNAREs is listed according to its structural classification as Q- or RSNARE.
The Q-SNAREs have been further divided as Qa-, Qb- and Qc-SNAREs (7) with one of each type of SNARE being required for a functional SNAREpin. Intracellular location (PM-plasma membrane, ER-endoplasmic reticulum) is shown: Golgi-vacuole denotes those
SNAREs localized to the prevacuolar compartment but also to Golgi or vacuole. The four SNAREs not anchored via a transmembrane
segment are designated with an asterisks. The number of SNARE motifs is shown; note Sec9 and Spo20 have two motifs and fulfill the
requirement of both Qb- and Qc-SNARE within the plasma membrane SNAREpin. The approximate size of the N-terminal domain proximal
to the SNARE motif is indicated with references for the functional and/or structural analyses of the N-terminal domains given

Name	Location	SNARE motifs	N-term domain	Function of N-terminal domain	References
Qa					
Ufe1	ER	1	253	SM (Sly1) recruitment	(54)
Sed5	Golgi	1	248	SM (Sly1) recruitment	(54,59)
Tlg2	Golgi	1	242	SM (Vps45) recruitment	(55,56)
Pep12	Golgi-vacuole	1	194	SM (Vps45) recruitment	(53)
Vam3	vacuole	1	185	SM (Vps33) recruitment	(67)
Sso1	PM	1	188	SM (Sec1) recruitment	(68,69)
Sso2	PM	1	193	SM (Sec1) recruitment	(68,69)
Qb					
Sec20	ER	1	201	Tip20 recruitment.	(64)
Bos1	ER-Golgi	1	150	?	
Gos1	ER-Golgi	1	133	?	
Vti1	Golgi-vacuole	1	124	?	(70)
Sec9*	PM	2	432	?	
Spo20*	PM	2	178	?	(62)
Qc					
Slt1	ER	1	148	Frq1 recruitment?	
Sft1	Golgi	1	4	None	
Bet1	Golgi	1	50	?	
Tlg1	Golgi	1	130	?	
Syn8	Golgi-vacuole	1	163	Glc7 recruitment?	
Vam7*	vacuole	1	247	PHOX domain, PI-binding	(50)
Sec9*	PM	2	432	?	
Spo20*	PM	2	178	?	(62)
R					
Sec22	ER-Golgi	1	128	?	(58)
Ykt6*	Golgi-vacuole	1	135	Regulation/sorting of Ykt6?	(49,60)
Nyv1	Golgi-vacuole	1	160	?	
Snc1	PM-vesicles	1	23	None?	
Snc2	PM-vesicles	1	22	None?	

**Table 1 Comparative analysis of Sec22 orthologs of different species**	**Query coverage**	**E value**	**Max Identity (%)**	**NCBI Reference number**
Fusarium graminearum	100	6e-159	100	XP_011323737.1
Alternaria alternata	100	7e-154	97	KIL91621.1
Saccharomyces cerevisiae	99	3e-67	46	NP_013370.1
Schizosaccharomyces pombe	100	1e-82	55	NP_596218.2

Trichoderma harzianum	100	1e-124	79	KKP06674.1
Sclerotinia sclerotiorum	100	2e-122	75	XP_001590003.1
Neurospora crassa	100	4e-116	75	XP_960888.1
F. verticillioides	100	3e-152	96	XP_018749557.1
Arabidopsis thaliana	98	5e-48	40	NP_172653.1
Drosophila melanogaster	96	3e-37	37	NP_001259103.1
Plasmodium falciparum	86	6e-28	31	XP_001351271.1
Neonectria ditissima	100	4e-136	84	KPM41255.1
Cordyceps confragosa	100	1e-129	80	OAA75842.1
Botrytis cinerea	100	3e-122	75	CCD48716.1
Colletotrichum gloeosporioides	100	2e-128	80	EQB50290.1
Homo sapiens	75	9e-39	43	XP_016858345.1
Caenorhabditis elegans	97	1e-34	34	NP_508198.1
Glycine max	98	8e-44	37	XP_003547819.1
Mus musculus	100	5e-45	39	NP_035472.1
